# Clinical profile of *FTL3*-ITD mutation in West Algerian population with acute myeloid leukemia

**DOI:** 10.22099/mbrc.2024.49437.1955

**Published:** 2024

**Authors:** Fatima Zohra Moghtit, Wefa Boughrara, Samia Dorgham, Ilies Koriche, Imene Ouahba, Amina Ammour, Amine Bekadja, Meriem Samia Aberkane

**Affiliations:** 1 Department of biology, Faculty of Science and Technology, University of Ain Temouchent-Belhadj Bouchaib-, Algeria; 2 Service de Cytogénétique et de Biologie moléculaire de l’Etablissement Hospitalo-universitaire d’Oran (1er Novembre); 3 École Supérieure en Sciences Biologiques d'Oran (ESSBO), BP 1042, Saim Mohamed 31003, Oran, Algeria; 4 Université des sciences et de la technologie d’Oran Mohamed Boudiaf; 5 Service d’hématologie et de thérapie cellulaire de l’Etablissement Hospitalo-universitaire d’Oran (1er Novembre); 6 Département de pharmacie, Faculté de Médecine, Université Oran1

**Keywords:** FLT3, ITD, Acute myeloid leukemia, Prevalence, West of Algeria

## Abstract

Acute myeloid leukemia (AML) is a cancer of the myeloid line of blood cells, characterized by the abnormal and rapid growth of cells. The mutation of the Fms-like tyrosine kinase 3 ligand gene (*FLT3*-ITD) represents an important factor in the prognosis of AML. The objective of this study was to determine for the first time the prevalence of *FLT3*-ITD mutation in west Algerian AML patients. A total of 160 AML patients were genotyped for *FLT3*-ITD mutation by using polymerase chain reaction. *FLT3*-ITD mutation was detected in 13% of patients. Mutation rates show no significant difference in the distribution of sex and age. A positive association was found between this mutation and a higher leukocyte and blast cells counts. We also found that the M3 and M5 subtype were the commonest in the *FLT3* mutated group. This preliminary study provides first-time prevalence estimates for *FLT3*-ITD mutation in acute myeloid leukemia patients from the West region of Algeria.

## INTRODUCTION

The acute myelogenous leukemia (AML) represents approximately 80% of acute leukemias in adults and 20% of those in children. It is responsible for 1.5% of tumor-related deaths and accounts for 4 new cases /100,000 inhabitants per year [1].

Many chromosomal abnormalities involved in AML are recognized as being good prognosis markers. Commonly observed are reciprocal translocations: t (8;21), t (15;17), and pericentric inversion of chromosome 16. However, other rearrangements are associated with a poor prognosis such as a deletion in chromosome 5 or 7 (not specific AML type) [2, 3]. In recent years, *FLT3* has been a subject of several studies as prognostic marker in AML patients. 

The Fms-like tyrosine kinase 3 (*FLT3*) gene, also named *FLK-2* (fetal liver kinase 2) or *STK-1* (human stem cell kinase 1), is located on 13q12 and consists of 24 exons [4] . It encodes a class III receptor tyrosine kinase (RTK III) and is expressed by stem cells, hematopoietic progenitors cells, brain, placenta, and liver [5]. The FLT3 receptor plays an important role in proliferation, differentiation **, **and survival of hematopoietic progenitors cells, in synergy with several other growth factors [6, 7]**. **Hence, *FLT3* is one of the most frequently altered genes in AML. The Internal Tandem Duplications of the *FLT3* gene (*FLT3*-ITDs) were identified as the most commonly occurring mutations in patients with AML [8]. These duplications induce constitutive auto-phosphorylation of the receptor in the absence of its ligand and initiates a series of signaling events which leads to an uncontrolled cell proliferation [9, 10]**. **The signaling properties of a FLT3 receptor with an ITD mutation differ from those of the wild type receptor in manner that clearly contributes to the process of leukemogenesis. STAT and FOXO transcription factors are abnormally activated in response to FLT/ITD signaling. Microarray studies have identified upregulation of the *Pim1* and *Pim2* proto-oncogenes, as well as interaction with the Wnt signaling pathway in FLT/ITD leukemia cells. Finally, c/EBP alpha, a transcription factor involved in myeloid differentiation, is downregulated by mutated receptors, indicative of the differentiation block that characterizes leukemia cells [10]. The frequency of *FLT3*-ITD mutation varies according to the type of leukemia: it is found preferentially in AML [8, 11, 12] but also detected at lower frequencies in myelodysplastic syndromes (MDS).

According to the literature, The *FLT3*-ITD mutation frequency varies among different ethnic populations. A relatively low frequency is identified in African populations (15-20%) [13-15] compared with other populations (20-30%) [16-18].

Indeed, mutation profiling of AML patients is part of the routine diagnostic workup for patients with de novo and recurrent AML. In Algeria, *FLT3* mutation profiling is not routinely performed in most public institutions. Therefore, there is little epidemiological data on the prevalence and heterogeneity of *FLT3* mutations and their clinical impact in Algerian AML patients. The purpose of this study is to describe the frequency of *FLT3*-ITD mutation in relation to patient demographics and specific AML classifications. Genotypic analysis of this mutation in a group of Algerian population would help and thus guide to devise precise therapeutic intervention in such patients.

## MATERIALS AND METHODS

**Patients: **A total of 160 patients from the West of Algeria diagnosed with AML, according to WHO criteria, were included in this study. These patients were referred from hematology department of the hospital-university of Oran to cytogenetic and molecular biology laboratory of EHU d’Oran during the period of January 2014 to June 2022. Previously untreated patients were included and patients with a history of chemotherapy/radiation therapy and patients with secondary AML were excluded. Clinical data were collected by reviewing medical records. The sampling and studies were conducted after obtaining written informed consent and the study was approved by the ethics committee. 

**Genotyping: **DNA was isolated from peripheral white blood cells by Maxwell® 16 Genomic DNA Purification Kit. The *FLT3*-ITD mutation was genotyped by polymerase chain reaction (PCR) method. The amplification was accomplished with a total 50 μl reaction mixture containing 1.5mM of MgCl_2_, 200 μM of dNTP, 2.5U Taq polymerase, 0.5 μM of each primer (forward: 5’-CAATTTAGGTATGAAAGCCAGC-3’; reverse: 5’CTTTCAGCATTTTGACGG CAACC-3’) and 50ng of genomic DNA. The PCR cycling conditions were as follows: 95°C for 09 min followed by 25 cycles of 95°C for 30 seconds, 57°C for 1 min, 72°C for 2 min, and a final elongation at 72°C during 10 minutes.

**Statistical analysis: **The demographic characteristics of AML are expressed as the mean ± standard deviation (SD) whereas qualitative variables are presented as numbers and percentages. The comparison of qualitative data between *FLT3*^-^ITD ^–^ and *FLT3*^-^ITD^+^ were evaluated using Chi-square test or Fisher’s exact test .p<0.05 was considered to be of statistical significance.

## Results

We enrolled in this study a total of 160 AML patients from the West of Algeria. Seven samples were excluded from the analysis due to unknown genetic profiles. Out of 153 studied cases, 87 were males and 66 were women. The mean age was 37.35 ±13.47 years. Details of demographic and clinical data are illustrated in Table 1.

**Table 1:** Demographics and clinical details of AML patients

**Variables**	**Total (n=153)**	**Male (n=87)**	**Female (n=66)**
**Age (years)** **mean±SD**	n=138 37.35 (±13.47)	n=78 38.57±13.84	n=60 35.51±13.08
**Leukocytes** **(x10** ^9^ **/L)**	n=128 3278262.4 (±3191801.9)	n=76 3403371.4 (±5798593.4)	n=53 3225687.4 (±5560118.4)
**Blast (%)** **mean±SD**	n=118 62.48 (±26.84)	n=66 63.54 (±27.28)	n=52 60.98 (±26.23)
**FAB type**	153	87	66
**M0**	4	4	0
**M1**	5	2	3
**M2**	9	7	2
**M3**	8	5	3
**M4**	13	7	6
**M5**	13	10	3
**M6**	6	3	3
**M7**	1	0	1
**Unknown**	94	49	45

n: Number, %: Percentage, SD: Standard deviation, M0:acute myeloblastic leukemia – minimally differentiated), M1: acute myeloblastic leukemia – without maturation, M2: acute myeloblastic leukemia –with granulocytic maturation, M3: Acute promyelocytic leukemia, M4: Acute myelomonocytic leukemia, M5: Acute monocytic - monoblastic leukemia, M6: Acute erythroleukemia, M7: Acute megakaryoblastic leukemia*.*

**Figure 1:**
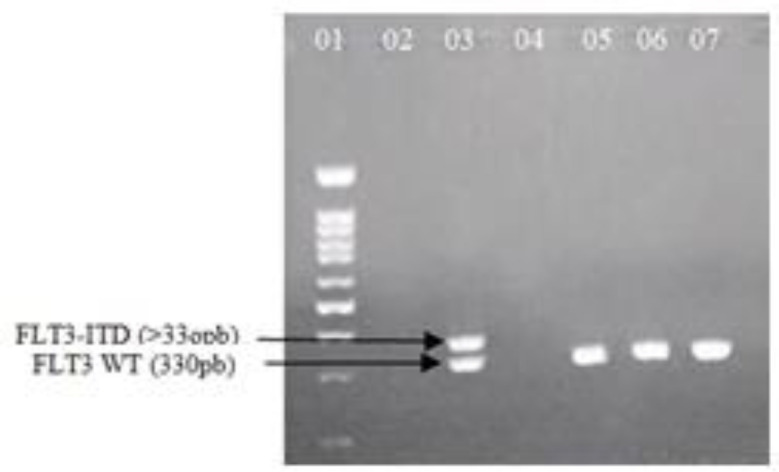
Representative picture of agarose gel showing wild type and *FLT3*-ITD mutant bands. Lane 01:100pb DNA Ladder, Lane02: Negative control, Lane 03: patient sample with *FLT3*-ITD mutation. Lanes 05-07: patients samples negatives for ITD mutation.

The PCR amplicon of ITD mutation in the *FLT3* showed different sizes of PCR products The 328 bp fragment indicates the size of the wild-type *FLT3* gene in the absence of ITD , while a positive result gave a band larger than 328 bp, indicating the presence of the *FLT3*-ITD mutation. A representative gel is shown in Figure 1.

As presented in the table 2, the *FLT3*-ITD mutation was detected in 21 patients (13.7%). There was no statistically significant difference in the distribution of sex and age according to *FLT3*-ITD mutation status. However, this mutation was found to be associated with hyperleuckocytosis and high blast percentage. Among FAB subtypes, *FLT3-*ITD mutation was more commonly reported within the M3, M5 subtype (Table 2).

**Table 2:** Distribution of *FLT3*-ITD mutations according to patient characteristics.

	**Total**				**Wild type ** ***FLT3***	**Muted ** ***FLT3*** **-ITD**	**P-value**
	153				132 (86.2)	21 (13.7)	
** Age groups n** **(%)**							
<25	31				29 (21.9)	2 (9.5)	0.39
26-50	95				81 (61.3)	14 (66.6)	
>50	27				22 (16.7)	5 (24)	
Sex n (%)							
Male	87				75 (57)	12 (57)	0.97
Female	66				57 (43)	9 (43)	
**Leukocytes mean±SD**							
1-4999	n=53 2228.6(±1379.3)				n= 50 2181.4(±1376.9)	n=3 3015(±1425.5)	
5000-10000	n=15 7120.6(±1267.09)				n=14 7272.1(±1165.4)	n=1 5000	
<10000	n=60 7545767.4 (±1050860.5)				n=47 8623359.4 (±1230523.5)	n=13 61746924 (±6160469.4)	0.03
Unknown	n=25				n=21	n=4	
Blast n (%)							
1-50	60				57(43.2)	3 (14.3)	
50-100	58				43 (32.6)	15 (71.4)	0.001
Unknown	35				32 (24.2)	3 (14.3)	
FAB type n (%)							
M0	4				4 (3)	0(0)	0.41
M1	5				5 (3.8)	0(0)	0.36
M2	9				9 (6.8)	0(0)	0.21
M3	8				5 (3.8)	3 (14.3)	0.04
M4	13				10 (7.6)	3 (14.3)	0.3
M5	13				9 (6.8)	4 (19)	0.06
M6	6				5 (3.8)	1 (4.8)	0.82
M7	1				1 (0.75)	0(0)	0.69
Unknown	94				84 (63.7)	10(47.6)	/

n: Number, %: Percentage, SD: Standard deviation, M0: acute myeloblastic leukemia – minimally differentiated), M1: acute myeloblastic leukemia - without maturation, M2: acute myeloblastic leukemia – with granulocytic maturation, M3: Acute promyelocytic leukemia, M4: Acute myelomonocytic leukemia, M5: Acute monocytic - monoblastic leukemia, M6: Acute erythroleukemia, M7: Acute megakaryoblastic leukemia.

## DISCUSSION

The acute myeloid leukemia is a heterogenous blood cancer which arises through the accumulation of genetic and epigenetic mutations. The *FLT3*-ITD mutation is the most frequent alteration in AML, found in approximately 25% of all cases [19].

The incidence of *FLT3*-ITD mutation in AML varies across different ethnicity. However, no data is available in Algerian patients. Currently, this is the first study to assess the prevalence of *FLT3*-ITD in AML patients from Western Algeria. Furthermore, several studies have reported that the presence of this mutation is associated with a poor prognosis; thus, *FLT3*-ITD genotyping has become an essential tool to identify those who may benefit from different targeted treatment options. Therefore, this study could help to choose the optimal treatment for AML patients. 

 The FLT3 is a transmembrane ligand-activated receptor tyrosine kinase. The *FLT3*-ITD mutation leads to constitutive activation of the FLT3 Kinase which plays a key role in cell proliferation and survival of leukemic cells [7]. In fact, *FLT3*-ITD is one of the most common mutations found in AML adult patients, especially in those with a normal Karyotype. It is often associated with a high leukaemic burden which conduce a poor prognosis, and has a negative effect on the management of AML patients [16, 17]. The first generation of multitargeted tyrosine kinase inhibitors (TKIs) drug have a poor response in AML patient with *FLT3*-ITD [20, 21].

In the present work, we investigated the *FLT3*-ITD mutation status in 160 AML patients. The mean patient age at the time of testing was 37.35±13.47, ranging from 16 to 68 years. This was a youthful age compared to other populations [22-26]. These findings may be explained by the young population structure in Algeria. On the other hand, Male to female ratio was 1.3:1 which is similar to that reported by Ayachi et al. in Eastern Algeria [27] and also in most countries [28, 29]. This male predominance is poorly understood and could be attributed to the high exposure to work-related and environnemental risk factors for this cancer.

In our study population, the results showed a frequency of *FLT3*-ITD mutation of 13.7%. In comparing this frequency to previous published data of different ethnic groups, this frequency is similar to those reported in Saudian [30], Egyptian [31], South African [13], Malysian [32] and Pakistanian populations [29, 33]. On the contrary, the frequencies observed in Germany [17], Thai [18], Chinese [34], Mexican [35], Iranian [36] and Indonesian populations [37] do not agree with our findings. On the other hand, no significant differences were found between FLT3 mutated and wild-type patients in terms of age and sex. This is in accordance with the findings of other studies [18, 31, 34]. However, Kondo et al reported a very low frequency of *FLT3*-ITD mutation in patient younger than 10 years [38].

Clinically, the results showed that AML patients with *FLT3*-ITD mutation had higher leucocytes count and an increased blast cell percentage at diagnosis compared with non mutated patients. These findings were consistent with some previous reports [39-40] and suggesting that this mutation may confer a survival advantage and increased proliferation of leukemia cells. Regarding FAB classification, this study, in accordance with others [16, 41], revealed that *FLT3*-ITD mutation occurs most commonly in M3 and M5 subtype. Otherwise, a lower frequency in M6 and M7 were reported in previous studies [16, 19, 41]. The difference in results between the current study and the previous studies can be explained by several factors such as ethnicity, patient selection criteria, genetics heterogeneity in the pathogenesis of AML and the number of patients included in different studies. 

The strength of our work relies in the study of the genetic profile of third world populations in general and Algerian population in particular. In addition, this study will help to provide a platform for future diagnosis and treatment of patients with AML. However, several limitations of our study need to be mentioned: A limited number of patients and limited hematological parameters were evaluated. In addition, other biomarker such as NPM1 mutations were not evaluated.

To the best of our knowledge, this is the first study to report the prevalence of *FLT3*-ITD mutation in our population. We identified 13% of patients harboring the mutation, which is similar to results published previously. Furthermore, the knowledge of the mutational profile of *FLT3* in a population may serve a crucial role in guiding treatment decision of AML. So, these findings need to be evaluated in a larger cohort of AML patients.

## Conflict of Interest:

All authors declare that there is no conflict of interest no relevant fnancial or nonfnancial interests to disclose.

## Authors’ Contribution:

Conceptualization: MSA, SD, WB; Methodology: FZM, IK, IO; Formal analysis and investigation: BW; Writing: original draft preparation: BW; Writing: review and editing: FZM; Supervision: MSA.
